# The association between age and accelerometry-derived types of habitual daily activity: an observational study over the adult life span in the Netherlands

**DOI:** 10.1186/s12889-018-5719-8

**Published:** 2018-07-04

**Authors:** Kimberley S. van Schooten, Jaap H. van Dieen, Mirjam Pijnappels, Andrea B. Maier, Alex J. van ‘t Hul, Martijn Niessen, Rob C. van Lummel

**Affiliations:** 1 Neuroscience Research Australia, University of New South Wales, Sydney, Australia; 2 Department of Biomedical Physiology and Kinesiology, Simon Fraser University, Burnaby, Canada; 3 Centre for Hip Health and Mobility, University of British Columbia, Vancouver, Canada; 4 Department of Human Movement Sciences, Vrije Universiteit Amsterdam, Research Institute Amsterdam Movement Sciences, Amsterdam, The Netherlands; 5 Department of Medicine and Aged Care, @Age, Royal Melbourne Hospital, University of Melbourne, Melbourne, Australia; 6 Department of Pulmonary Diseases, Radboud University Medical Center, Nijmegen, The Netherlands; 7 McRoberts BV, The Hague, The Netherlands

**Keywords:** Health, Aging, Sedentary behaviour, Physical activity, Mobility

## Abstract

**Background:**

Advances in sensor technology allow for objective and high-resolution monitoring of physical activity and sedentary behaviour. Novel epidemiological data is required to provide feedback on an individual’s habitual daily activity in comparison to peers and might eventually lead to refined physical activity guidelines.

**Methods:**

We merged data of 762 people between 18 and 99 years of age, who all wore a DynaPort MoveMonitor accelerometer on their lower back during 1 week in daily-life, to provide insight into habitual types and durations of daily activities, and examine the association between age and physical activity and sedentary behaviour.

**Results:**

We found associations between age and almost all activity outcomes. These associations suggested that physical activity declines and sedentary behaviour increases from the age of 50. We further describe an association with gender, with men walking more often in fewer but longer bouts and having fewer, longer bouts of sitting and standing.

**Conclusions:**

These data provide a valuable reference and may call for more age- and gender-specific activity interventions.

**Electronic supplementary material:**

The online version of this article (10.1186/s12889-018-5719-8) contains supplementary material, which is available to authorized users.

## Background

Exercising regularly and limiting sedentary behaviour are associated with health benefits over the entire life span. There is strong epidemiological evidence that regular physical activity is related to a decreased risk of cardiovascular disease, mobility decline, and early mortality [1, 2]. Moreover, recent studies provide evidence that regular moderate-to-vigorous intensity physical activity is important for maintaining mental health and cognition [3, 4]. Prolonged sedentary behaviour, such as sitting or lying, has negative health effects even in people who are considered physically active [5–7]. Hence, sufficient physical activity and limited sedentary behaviour is important to maintain health.

Despite the clear health benefits of physical activity, 31% of adults, and 40 to 50% of people aged 60 years and older, report to not attain the recommended levels of 30 min of moderate-intensity physical activity on at least 5 days every week, 20 min of vigorous-intensity physical activity on at least 3 days every week, or an equivalent combination of 600 metabolic equivalent minutes per week [8]. Previous studies show that higher age is associated with less time spent in moderate-to-vigorous-intensity physical activity, and more time spent sedentary [9–13]. It thus seems that with increasing age, people tend to become less physically active, which may adversely affect their physical function, and hence their capacity and motivation to engage in physical activities. This vicious circle can be prevented or remediated by regular and sufficient physical activity [14].

Information on habitual levels of physical activity and sedentary behaviour is required for the development of physical activity guidelines and personalized advice. Current physical activity guidelines are mostly based on evidence from self-reported activity levels. While people tend to be accurate in self-reports of exercise, they are less accurate in recalling their activities of daily living [15]. This may be enhanced in people with impaired cognition and limited physical activity levels, as often coincide with ageing. Objective assessment of physical activity using body-worn inertial sensors may provide more accurate information. Previous studies generally employed uniaxial accelerometers and extracted activity counts [9–13], which has the downside that information about the type of performed activity is lost. Advances in sensor and processing technology now allow for activity recognition, identifying not only the intensity of the performed activity, but also the type of activity. Insight in the type of daily activities can provide more detailed understanding of the origin of differences or changes in habitual activity, and may enrich personalized advice and physical activity guidelines by making these more specific.

This paper describes how the habitual types of physical activity and sedentary behaviour associate with age, to provide more detailed insight into the variation in physical activity across the lifespan.

## Methods

### Participants

**Fig. 1:**
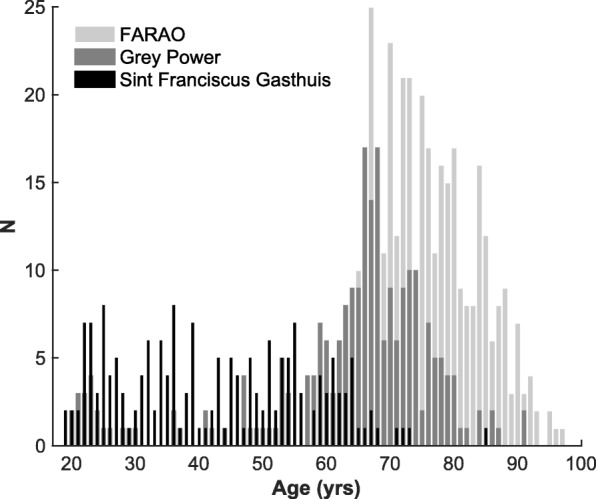
Distribution of age of the participants stratified by cohort

We merged data of 762 participants of the FARAO, Grey Power and Sint Franciscus Gasthuis studies (Fig. 1). These studies used the same protocol to measure physical activity and sedentary behaviour during daily life, i.e. by means of a trunk-worn tri-axial accelerometer attached centrally over the lower back with an elastic belt, but their study periods and inclusion criteria differed as detailed below.

We included 350 people from the FARAO study [16]. The FARAO cohort consisted of people between the ages of 65 and 99 years without major cognitive or physical impairments as assessed by a mini mental state examination score [17] exceeding 18 points and the ability to walk at least 10 m, with or without a walking aid. Participants were recruited between March 2011 and January 2014 in Amsterdam (The Netherlands) and surroundings via general practitioners, pharmacies, training groups, hospitals, and residential care facilities. All participants provided written informed consent and the protocol was approved by the medical ethical committee of the VU University Medical Center, The Netherlands.

We included 233 people from the Grey Power study [18]. The Grey Power cohort consisted of people aged 18 years and older, and had no further exclusion criteria. Participants were recruited in November 2014 during the Grey Power debate events at the VU University Medical Center in Amsterdam (The Netherlands). These debates were freely accessible for the general population to promote healthy ageing and attracted a predominantly vital and motivated group of socially active community-dwelling participants. All participants provided written informed consent and the protocol was approved by the medical ethical committee of the VU University Medical Center, The Netherlands.

We included 179 people from the Sint Franciscus Gasthuis study [19]. The Sint Franciscus Gasthuis cohort consisted of people between the ages of 18 and 85 years, who had no medical conditions that would impair physical activity and who did not participate in strenuous competitive sport activities. Participants were recruited between December 2013 and March 2014 in The Hague and Rotterdam (The Netherlands) and their surroundings. The medical ethics committee of the Sint Fransiscus Gasthuis considered the study outside the remit of the medical research involving human subjects act (Dutch WMO act) and provided a written confirmation that the need for approval was waived.

### Physical activity and sedentary behaviour assessment

Participants wore a tri-axial trunk accelerometer (DynaPort MoveMonitor, McRoberts, The Hague, The Netherlands) on their lower back for a period of 1 week. The accelerometer had a sample frequency of 100 samples/s and a range of -6 g to + 6 g. Participants were instructed to wear the accelerometer at all times, except during aquatic activities such as showering since this would damage the device. Bouts of non-wearing, walking, cycling, standing, sitting and lying were identified using a commercially available algorithm (MoveMonitor, McRoberts). In general, this algorithm first determines whether the accelerometer was worn based on threshold and frequency analysis of the raw signal [20], and subsequently differentiates between high intensity (walking and cycling) and lower intensity (sitting, lying, and standing) activities [21, 22]. Sitting, lying and standing bouts could have any duration, walking bouts required at least 3 steps to be taken, and cycling bouts had a minimum duration of 1 min. The accuracy of this algorithm has been shown to be good in both older and younger populations [23–26]. Recent validation studies report accuracies of 88% for non-wearing [27] and accuracies of 86–99% for walking, 89% for cycling, 88–97% for standing, 91–99% for sitting, and 97% for lying [22, 25]. Data of days on which the accelerometer was worn more than 75% of the time, i.e. at least 18 h, were averaged to determine habitual type of daily activity and a minimum of 4 valid wearing days was required for a participant’s data to be included in the analysis [28]. We determined how age associated with habitual daily activity in terms of the following 9 outcomes: movement intensity, the total duration of walking, cycling, standing, sitting and lying, and the number of walking and sitting bouts, and maximum duration of walking bouts.

### Statistical analysis

Visualisation of the relation between daily activity and age showed a clear non-linear relation for most activity outcomes. Hence, age was split into bins of 18–30, 31–40, 41–50, 51–60, 71–80 and 81–99 to test for non-linear associations while ensuring adequate group size (minimum of 38 people per group, see Table 1). We first compared the participant demographics between the age groups using an ANOVA with post-hoc *t*-tests or Chi-square tests with post-hoc *z*-tests. All post-hoc tests were Bonferroni corrected. Subsequently, we ran generalized linear model (GLM) regressions with age as a categorical predictor and the activity outcome as continuous variables to determine the association between age and physical activity and sedentary behaviour with correction for participant demographics that differed between the age groups and that may influence activity levels. For each age group, we used the previous age group as reference to identify onsets of change in activity levels. All data processing and statistical analysis were performed in Matlab 2015a (Mathworks, Natrick, USA).

**Table 1:** Participant demographics for each age group

Age group (yrs)	N	Age (yrs)	Height (cm)	Weight (kg)	Gender (% men)	Eligible data (days)
18–30	62	23.8 (2.6)	173.3 (10.3)^a^	69.9 (13.1)	29.0%	5.8 (0.7)
31–40	47	34.8 (2.8)	174.8 (9.5)^a^	74.2 (13.7)	21.3%^b^	5.8 (0.6)
41–50	38	45.3 (2.7)	171.3 (8.9)	76.2 (13.2)	29.0%	5.8 (0.5)
51–60	64	55.0 (2.9)	171.0 (8.9)	76.2 (14.5)	21.9%^b^	5.8 (0.6)
61–70	185	65.6 (2.5)	171.2 (9.2)	75.4 (15.5)	37.8%	5.9 (0.5)
71–80	236	74.1 (2.9)	169.5 (8.8)	74.0 (14.3)	39.4%	5.9 (0.5)
81–99	130	85.1 (4.1)	168.2 (9.5)^a^	72.0 (12.0)	46.9%^b^	5.8 (0.5)

All values are mean (SD)

^a^people in the 81–99 year-group were significantly shorter than people in the 18–30- and 31–40-year groups

^b^people in the 81–99-year group were significantly more often men than people in the 31–40- and 51–60-year groups

## Results

The age groups differed significantly in their gender distribution (*χ*^*2*^ (6, 762) = 20.16, *p* = 0.003) and height (*F* (6, 739) = 4.52, *p* = 0.0002). The proportion of men was significantly higher in the 81–99-years group compared to the 31–40- and 50–61-year groups (*z*-tests with Bonferroni correction; all *p* < 0.05; Table 1). Moreover, people in the 81–99 years group were on average respectively 5.0 and 6.5 cm shorter than people in the 18–30- and 31–40-year groups (*t*-tests with Bonferroni correction; *p* < 0.01; Table 1). Subsequent analyses were corrected for gender but not height, since height was not associated with habitual daily activity, and was considered inherent to the trend of increasing adult height over generations and decreasing height during the ageing process. The average duration that participants wore the activity monitor was 23.60 (SD 0.42) hours per day. The number of eligible measurement days was akin for the age groups (*F* (6, 755) = 0.29, *p* = 0.95).

**Fig. 2:**
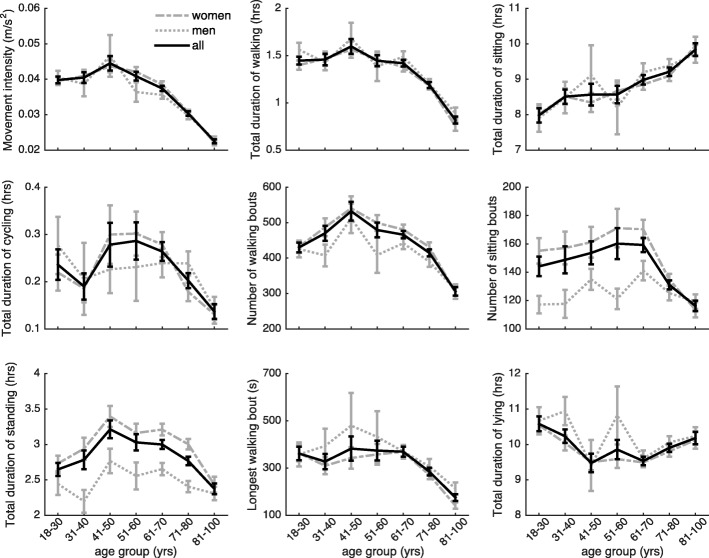
The relation between age and physical activity and sedentary behaviour

Age group had a clear association with most daily activity outcomes (Fig. 2). People in the 41–50-year group had a significantly higher movement intensity, lower total lying duration, and longer total standing duration compared to people in the 31–40-year group (Table 2). They further exhibited more bouts of walking with a similar total duration, which indicates that their average walking bouts were shorter, despite similar maximum walking bout duration. People in the 51–60- and 61–70-year groups had significantly lower movement intensity compared to people in the 41–50- and 51–60-year groups, respectively. People in the 71–80-year group had significantly lower movement intensity, lower total walking, cycling and standing duration, and fewer bouts of walking with a lower maximum bout duration compared to people in the 61–70-year group. They furthermore had fewer bouts of sitting with a similar total duration, which indicates that their sitting bouts were on average longer. People in the 81–99-year group had again a significantly lower movement intensity, longer duration of lying or sitting, with fewer, and thus longer sitting bouts, lower duration of cycling and lower total duration of walking, with shorter and fewer walking bouts compared to people in the 71–80-year group.

Over all age groups, men exhibited fewer bouts of sitting and walking, and had a lower total duration of standing compared to women. They further had a significant longer total duration of walking and maximum walking bout duration (Table 2).

**Table 2:** The association of age with habitual daily activity corrected for gender

	Age group (years)							Male gender
	18–30	31–40	41–50	51–60	61–70	71–80	81–100	
Movement intensity (m/s^2^)	0.0401 (0.0012)	0.0407 (0.0018)	0.0448 (0.0020)^a^	0.0411 (0.0019)^a^	0.0378 (0.0013)^a^	0.0308 (0.0009)^b^	0.0230 (0.0010)^b^	− 0.0011 (0.0007)
Physical activity								
Total duration of walking (hrs)	1.43 (0.06)	1.44 (0.09)	1.58 (0.10)	1.43 (0.09)	1.39 (0.07)	1.16 (0.04)^b^	0.78 (0.05)^b^	0.07 (0.03)^a^
Bouts of walking (n)	440 (19)	477 (28)	542 (32)^a^	486 (30)	478 (21)	428 (14)^b^	322 (16)^b^	− 33 (11)^b^
Maximum walking bout duration (s)	349.99 (32.83)	319.05 (49.28)	370.64 (55.58)	365.27 (52.17)	353.96 (37.06)	269.06 (25.01)^b^	156.18 (27.85)^b^	40.46 (19.43)^a^
Total duration of cycling (hrs)	0.23 (0.03)	0.19 (0.05)	0.28 (0.05)	0.28 (0.05)	0.26 (0.04)	0.20 (0.02)^a^	0.13 (0.03)^a^	0.01 (0.02)
Total duration of standing (hrs)	2.79 (0.11)	2.89 (0.16)	3.35 (0.18)^a^	3.14 (0.17)	3.18 (0.12)	2.96 (0.08)^a^	2.60 (0.09)^b^	− 0.48 (0.06)^b^
Sedentary behaviour								
Total duration of sitting (hrs)	7.94 (0.24)	8.48 (0.36)	8.53 (0.40)	8.54 (0.38)	8.93 (0.27)	9.16 (0.18)	9.77 (0.20)^a^	0.13 (0.14)
Bouts of sitting (n)	149 (7)	153 (11)	159 (13)	164 (12)	166 (8)	138 (6)^b^	125 (6)^a^	− 18 (4)^b^
Total duration of lying (hrs)	10.51 (0.23)	10.18 (0.34)	9.40 (0.39)^a^	9.80 (0.36)	9.44 (0.26)	9.80 (0.17)^a^	10.06 (0.19)	0.25 (0.14)

All values are beta (standard error). Statistical tests were against the previous age group

^a^indicates *p* < 0.05

^b^indicates *p* < 0.001

## Discussion

We investigated the relation between age and the habitual types of daily activities across a range of ages. Our results suggest that the durations of different types of physical activity and the intensity of these activities are relatively constant until the age of 50, after which a rapid decline occurs. Sedentary behaviour increases slightly later, starting around the seventh decade. These results agree with those of Schrack and colleagues [29], who showed in 611 people aged 32 to 93 that activity counts (most similar to our movement intensity) show a rapid decline after the fifth to sixth decade. The results further extend previous studies that reported that time spent in moderate-to-vigorous-intensity physical activity is lower with higher age [9–13], by revealing that this difference may be primarily attributed to a reduction of walking activity and increase in sitting duration (Cohen’s *d* effect sizes of − 1.17 for total walking duration, − 0.72 for number of walking bouts, − 0.68 for maximum walking bout duration, 0.72 for total duration and − 0.40 for number of sitting bouts, when comparing 81–99-year to 18–30-year group).

We further found a clear association between gender and habitual daily activities, with men walking more often in fewer but longer bouts and having fewer, and longer bouts of sitting and standing. These differences may explain the well-established gender difference in activity counts with men having higher counts than women [9–11, 30–32], and suggest that activity promotion programs may need to take gender into consideration. A secondary analysis of the data taking interactions between gender and age into account, suggest that except for the number of standing bouts, the association between gender and habitual daily activity depends on age and is more pronounced in the older age groups (Additional file 1: Table S1). However, since the number of men in each age group was relatively low (10 to 83 men per age group), replication in a larger sample is warranted.

We provided data on habitual levels of type of physical activity and sedentary behaviour to support the development of personalized advice and show the need to update physical activity guidelines to be aligned with objective monitoring of daily activity. Our findings indicate that, on average, people spend between 0.7 and 1.5 h walking per day with maximum bout durations between 2.6 and 6.2 min (i.e. 156.18 to 370.64 s). Since most physical activity guidelines recommend a minimum activity duration of 10 min for aerobic activities, such as walking, to be considered to contribute to their achievement (e.g. [33]), updates to match the increased resolution of wearable sensors, which detect short stops while e.g. waiting for a traffic light, seem desired. Comparison of individual levels of type of physical activity and sedentary behaviour to age-specific reference data can aid to formulate personalized advice.

This study is the first to investigate the association between age and types of daily activity using accelerometry obtained during full days in a large cohort, however it also has its limitations. First, we aggregated data of 3 independent studies, which despite similar inclusion criteria and identical methods may have included different populations. Inclusion of study as a covariate in our statistical analyses, however, did not change the trends with age. Second, the analysis was cross-sectional and generational differences may have affected our outcomes. Future longitudinal studies should aim to follow activity levels over longer time periods. Third, our exclusion criteria likely resulted in bias towards a healthy population, limiting generalisability to populations with medical conditions that may affect physical activity. Fourth, the activity monitor was not waterproof, which may have led to an underestimation of the amount of physical activity for some. Fifth, the activity classifications were based on an algorithm, which may or may not fit well for all ages. Even though the activity classification was found to be valid compared to observation in young and older adults [23, 26], as well as patient populations [24, 25, 27], previous studies suggest that the differentiation between sitting and standing deserves improvement [26]. Future studies should investigate the robustness of our results in different populations and with different activity classification algorithms.

## Conclusions

In sum, we provide data on objectively assessed habitual type of physical activities among age groups from 18 to 99 years. Our findings show that physical activity generally declines and sedentary behaviour increases with ageing from the age of 50 onwards. Our findings further suggest that these changes may be primarily due to a reduction in walking activity and increase in sitting duration. These data provide a valuable reference when evaluating an individual’s habitual daily activity and signal the need for aligning physical activity guidelines with objective monitoring of daily activity.

## Additional file

Additional file 1: **Table S1.** The association of age and gender with habitual daily activity. (DOCX 18 kb)
