# Supplementary material for: The association between age and accelerometry-derived types of habitual daily activity: an observational study over the adult life span in the Netherlands
Source: BMC Public Health. 2018 Jul 4;18:824. doi: 10.1186/s12889-018-5719-8 (PMC6031182; doi:10.1186/s12889-018-5719-8)
Supplement: Supplementary file 1 — Table S1. The association of age and gender with habitual daily activity. (DOCX 18 kb) [file 12889_2018_5719_MOESM1_ESM.docx]

**Additional file 1**

Table S1: **The association of age and gender with habitual daily activity**.

|  | **Age group (years)** | | | | | | | **Male gender** | **Interaction term** | | | | | |
| --- | --- | --- | --- | --- | --- | --- | --- | --- | --- | --- | --- | --- | --- | --- |
|  | **18-30** | **31-40** | **41-50** | **51-60** | **61-70** | **71-80** | **81-100** |  | **31-40 x male** | **41-50 x male** | **51-60 x male** | **61-70 x male** | **71-80 x male** | **81-100 x male** |
| Movement intensity (m/s2) | 0.0395 (0.0014) | 0.0409 (0.0021) | 0.0437 (0.0023) | 0.0421 (0.0022) | 0.0385 (0.0016)* | 0.0307 (0.0012)** | 0.0221 (0.0013)** | 0.0010 (0.0026) | -0.0033 (0.0042) | 0.0052 (0.0047) | -0.0086 (0.0043)* | 0.0028 (0.0031) | 0.0021 (0.0019) | 0.0017 (0.0020) |
| **Physical activity** |  |  |  |  |  |  |  |  |  |  |  |  |  |  |
| Total duration of walking (hrs) | 1.40 (0.07) | 1.47 (0.10) | 1.56 (0.12) | 1.46 (0.11) | 1.38 (0.08) | 1.18 (0.06)** | 0.75 (0.07)** | 0.16 (0.13) | -0.20 (0.21) | 0.15 (0.23) | -0.19 (0.21) | 0.19 (0.16) | -0.09 (0.09) | 0.12 (0.10) |
| Bouts of walking (n) | 432 (22) | 487 (33) | 540 (37) | 499 (35) | 481 (25) | 431 (18)* | 303 (21)** | -6 (41) | -73 (66) | 51 (74) | -62 (68) | 52 (49) | -2 (29) | 48 (32) |
| Maximum walking bout duration (s) | 363.34 (38.46) | 309.85 (56.90) | 342.67 (64.56) | 357.95 (60.92) | 368.98 (43.21) | 270.47 (31.95)* | 140.89 (37.39)** | -5.54 (71.37) | 89.26 (115.58) | 53.35 (128.80) | -63.12 (119.48) | -73.18 (86.28) | 36.10 (51.48) | 36.18 (56.25) |
| Total duration of cycling (hrs) | 0.22 (0.04) | 0.19 (0.05) | 0.30 (0.06) | 0.30 (0.06) | 0.28 (0.04) | 0.18 (0.03)* | 0.13 (0.03) | 0.05 (0.07) | -0.04 (0.11) | -0.09 (0.12) | 0.00 (0.12) | 0.03 (0.08) | 0.10 (0.05)* | -0.05 (0.05) |
| Total duration of standing (hrs) | 2.73 (0.13) | 2.94 (0.19) | 3.40 (0.21)* | 3.16 (0.20) | 3.21 (0.14) | 3.00 (0.10)* | 2.43 (0.12)** | -0.29 (0.23) | -0.46 (0.38) | 0.11 (0.42) | 0.02 (0.39) | 0.05 (0.28) | -0.04 (0.17) | 0.48 (0.18)* |
| **Sedentary behaviour** |  |  |  |  |  |  |  |  |  |  |  |  |  |  |
| Total duration of sitting (hrs) | 8.01 (0.28) | 8.52 (0.41) | 8.34 (0.46) | 8.67 (0.44) | 8.85 (0.31) | 9.10 (0.23) | 9.94 (0.27)* | -0.11 (0.51) | 0.08 (0.83) | 0.80 (0.93) | -1.23 (0.86) | 0.81 (0.62) | -0.06 (0.37) | -0.51 (0.41) |
| Bouts of sitting (n) | 155 (9) | 157 (13) | 161 (15) | 171 (14) | 170 (10) | 135 (7)** | 113 (8)* | -38 (16)* | -1 (26) | 13 (29) | -24 (27) | 21 (20) | 20 (12) | 15 (13) |
| Total duration of lying (hrs) | 10.55 (0.27) | 10.04 (0.39) | 9.50 (0.45) | 9.59 (0.42) | 9.49 (0.30) | 9.81 (0.22) | 10.13 (0.26) | 0.12 (0.50) | 0.79 (0.80) | -1.00 (0.89) | 1.33 (0.83) | -1.13 (0.60) | 0.15 (0.36) | -0.14 (0.39) |
|  |  |  |  |  |  |  |  |  |  |  |  |  |  |  |

All values are beta (standard error). Statistical tests were against the previous age group; ^*^ indicates *p* < 0.05 and ^**^ indicates *p* < 0.001.
